# Characterization of Acid Hydrolyzed Taro Boloso-I (*Colocasia esculenta* Cultivar) Starch as a Diluent in Direct Compression of Tablets

**DOI:** 10.1155/2024/6560070

**Published:** 2024-05-29

**Authors:** Solomon Gashaw, Afewerk Getachew, Fantahun Mola

**Affiliations:** ^1^Mekelle University, College of Health Sciences, School of Pharmacy, Department of Pharmaceutics, Mekelle, Ethiopia; ^2^Addis Ababa University, College of Health Sciences, School of Pharmacy, Department of Pharmaceutics and Social Pharmacy, Addis Ababa, Ethiopia

## Abstract

Corn, wheat, rice, potato, and cassava starches have been widely used as pharmaceutical excipients. However, the search for cost-effective local starch alternatives is necessary due to the availability and usage constraints. In Ethiopia, various plant species, including Taro Boloso-I, have been explored as potential sources of pharmaceutical starch. It is a variety of *Colocasia esculenta* with a high tuber yield and high starch content. However, the native starch requires modifications to enhance its functionality. Therefore, this study aimed to improve the native starch through acid modification and evaluate its performance as a direct compressible tablet excipient. The native starch was treated with a 6% w/v HCl solution for 192 hours, resulting in acid-modified Taro Boloso-I starch, which was then evaluated for suitability for direct compression. XRD patterns of both the native and modified starch showed characteristic A-type crystals, with significantly higher relative crystallinity observed in the latter. Additionally, the acid-modified starch exhibited a lower moisture content and improved flow properties. The compaction study also demonstrated its improved compactibility (tensile strength: 16.82 kg/cm^2^), surpassing both the native starch (13.17) and Starch 1500® (11.2). The modified starch also showed a lower lubricant sensitivity compared to the native starch and Starch 1500®. Furthermore, paracetamol tablets made with the modified starch exhibited higher mechanical strength and lower friability in all paracetamol concentrations. It incorporated up to 40% paracetamol while maintaining acceptable tablet characteristics, whereas the native starch and Starch 1500® were limited to 30% (w/w). Based on these findings, the modified starch showed promise as an alternative direct compressible excipient in tablet manufacturing.

## 1. Introduction

Naturally occurring polymers have been extensively used as pharmaceutical excipients for a significant period [1]. Among these polymers, starch stands out as a notable example. It is a biodegradable polysaccharide abundantly found in various plant organs [2]. The global production of starch is primarily based on sources such as corn, wheat, rice, potato, and cassava [3]. However, the challenge lies in harnessing locally available starches that offer cost-effective excipients with multifunctional properties [4]. In Ethiopia, various plant species such as enset [5], Dioscorea [6], Godare [7], cassava [8], Ethiopian potato [9], and Taro Boloso-I (TBI) [10] have been examined as potential starch sources for pharmaceutical applications.

**Figure 1:**
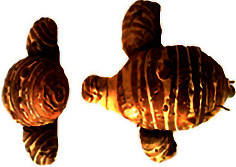
Taro Boloso-I plant tubers (photo taken by Solomon Gashaw).

TBI, an improved variety of *Colocasia esculenta*, was officially released by the Areka Agricultural Research Institute in Ethiopia [11]. With its higher cultivation yield and starch content, approximately 84% on a dry weight basis, the tuber of TBI (Figure 1) holds great promise as an alternative source of starch. However, it has limitations as a compressible tablet excipient due to its poor compressibility and flow properties [12]. Previous attempts to improve its functional characteristics have involved modifications such as acetylation and pregelatinization [12, 13]. However, acetylation proved to be a complex method, whereas pregelatinization did not effectively improve the compressibility. Consequently, a simple yet effective method is needed to enhance the compressibility and flow properties of native TBI starch (NTBIS).

Acid modification is one of the modifications that can be performed with relative ease. It involves the controlled addition of acid to an aqueous suspension of native starch [14]. Several studies have explored the potential of acid-modified starch as a compressible tablet excipient [15–21]. These studies have shown that the modification improved the flowability, compatibility, and dilution potential of native starches, making them suitable for direct compression (DC). These changes were suggested to be due to the change in size, shape, density, packing arrangements, and relative crystallinity of starch granules. These, in turn, affect the cohesiveness of their powder particles and the strength of intermolecular force during compression.

DC is a tablet manufacturing process that involves compressing powder mixtures without any prior treatment [22]. This method improves drug stability and reduces the number of unit operations, resulting in cost benefits. As a result, pharmaceutical manufacturers prefer it [23]. The success of a DC process is highly dependent on the characteristics of the excipients used such as the flowability, compactability, lubricant sensitivity, and dilution potential [16, 24]. Therefore, this study aims to modify NTBIS through acid modification and assess its suitability as a DC excipient in tablet formulations.

## 2. Materials and Methods

### 2.1. Materials

The TBI tubers were obtained from the Areka Agricultural Research Institute, located in Areka City (300 km south of Addis Ababa), Wolaita, Ethiopia. Hydrochloric acid BP (Loba Chemie Pvt. Ltd., India), sodium chloride (Abron Chemicals, India), and sodium hydroxide (Sisco Research Laboratories Pvt. Ltd., India) were of analytical grade and were purchased from the local market. Paracetamol powder BP (Anhui BBCA Likang Pharmaceutical Co. Ltd., China), magnesium stearate (Anhui Sunhere Pharmaceutical Excipients Co., Ltd., China), zinc stearate (Anhui Sunhere Pharmaceutical Excipients Co., Ltd. Ltd., China), sodium croscarmellose BP (Rossmell Industries, Phase II, G.I.D.C., India), potassium dihydrogen orthophosphate BP (Loba Chemie Pvt. Ltd., India), and disodium hydrogen orthophosphate dihydrate (TM Media, Titan Biotech Ltd., India) were donated by Addis Pharmaceutical Factory P.L.C. Starch 1500® (Colorcon, France) was kindly donated by the Department of Pharmaceutics and Social Pharmacy, School of Pharmacy, Addis Ababa University.

### 2.2. Methods

#### 2.2.1. Starch Extraction

The method described by Balcha et al. [10] was followed to extract the starch. Initially, fresh TBI tubers were washed, peeled, and cut into small pieces. The pieces were crushed with a blender machine (Sinbo, SHB-3088, China) using a 1% NaCl solution (w/v). The resulting mixture was then washed multiple times with a solution containing 1% NaCl and 0.03 N NaOH and filtered through a muslin cloth to eliminate cell debris. The sediment obtained was further washed with distilled water until the supernatant was clear and pH neutral. Finally, the starch was dried at 40°C in a hot air oven (Memmert SM-200, Germany), ground into a fine powder using a mortar and pestle, sieved through a 180 *μ*m mesh (FRESCH, Germany), and stored in a tightly sealed bottle for future use.

#### 2.2.2. Acid Modification

NTBIS was subjected to hydrolysis using HCl acid, following the procedure outlined in the study by Atichokudomchai et al. [15]. Initially, 400 grams of NTBIS was suspended in 600 ml of HCl solution (6% w/v) at room temperature for 192 hours. Subsequently, the suspension was neutralized with NaOH solution (10% w/v) and washed with distilled water until the pH became neutral (pH 7). Eventually, the acid-modified TBIS (AMTBIS) slurry was dried in a hot air oven (at 40°C for 24 hours), powdered, sieved (180 *μ*m sieve), and stored for future use.

#### 2.2.3. Spray Drying

Spray drying was carried out following the procedure described by Bilancetti et al. [25]. A 40% suspension of the sample was prepared and introduced into the drying chamber of the tall-form spray dryer (FT80, Armfield, USA), with the input and outlet temperatures set at 180 and 75°C, respectively. Subsequently, the spray-dried samples were sifted through a 180 *μ*m mesh sieve and stored for future use.

#### 2.2.4. Recovery Yield of the Acid Hydrolysis Process

Recovery yield was calculated as the percentage by comparing the weight of starches by dry weight before and after acid hydrolysis [26].

#### 2.2.5. X-Ray Diffraction Studies

The samples were analyzed using XRD patterns, following the method outlined by Nwokocha and Williams [27]. An X-ray diffractometer (XRD-7000, Shimadzu, Japan) was used in 2θ modes. The position of the peaks was determined using a Cu target tube set at 40 kV (30 mA) power, in the 5–50° range of 2θ, with a single crystal graphite monochromator. The degree of crystallinity was then calculated using equation (1), as described by Singh et al. [28],

(1) $$\begin{array}{c} \text{Crystallinity}\;\left(\%\right)=\frac{\text{Area of the crystalline portion}}{\text{Total area}}\times 100. \end{array}$$

#### 2.2.6. Density and Related Properties

50 gram starch powder was carefully poured into a 250 ml measuring cylinder, and the volume occupied was recorded. The measuring cylinder was then tapped 300 times on a tap densitometer (ERWEKA, D-63150, Germany), and the tapped volume was recorded. The bulk and tap density, Carr's index (CI), and Hausner's ratio (HR) were then calculated [29].

#### 2.2.7. Flow Rate and Angle of Repose

To measure the flow rate and the angle of repose, a powder flowability tester (PHARMA TEST, PTG-S4, Germany) was used. A 30 g sample of starch powder was placed into a stainless steel funnel with a 15 mm outlet nozzle. The funnel was positioned at a height of 10 cm above the base, which had a diameter of 10 cm. The powder was then allowed to flow completely through the outlet nozzle, and the machine recorded the output readings for the flow rate and angle of repose.

#### 2.2.8. Determination of Moisture Content

The moisture content of the starch samples was determined according to the method described by Olayemi et al. [30]. Two grams of sample was weighed on a dry Petri dish and placed in an oven at 130°C. Then, 2 hours later, the sample was removed, cooled, and weighed. The moisture content was then calculated as the percentage of weight loss before and after drying.

#### 2.2.9. Determination of Swelling Power and Solubility

The swelling power (SP) and the water solubility index (WSI) were determined as described by Odeku and Picker-Freyer [31]. 0.5 g starch suspensions in 10 ml of distilled water were prepared in centrifuge tubes and placed in a water bath (HH-S4, 20225101, Germany) at 25, 35, 45, 55, 65, 75, and 85°C. After 30 min, the test tubes were withdrawn, cooled, and centrifuged at 3000 rpm for 15 min. The supernatant was then decanted and dried in an oven at 120°C for 4 hours to a weight (*W*_1_). The weight of the residue (*W*_*R*_) was also determined, and then, the WSI and SP were calculated using the following equations:

(2) $$\begin{array}{c} \text{WSI}=\frac{W_{1}}{0.5}\times 100, \end{array}$$

(3) $$\begin{array}{c} \text{SP}=\frac{W_{R}\times 100}{0.5\times \left(100-\text{WSI}\right)}. \end{array}$$

#### 2.2.10. Determination of Moisture Sorption Pattern

The moisture sorption patterns of the samples were determined as described by Gebre-Mariam et al. [5]. First, five chambers of relative humidity (RH) (100, 75.6, 60, 40, and 20%) were prepared in a Pyrex desiccator at room temperature. Then, a two-gram sample (predried at 120°C for 4 h) was placed on dry Petri dishes and kept in the chambers. After seven days, the Petri dishes were removed and the moisture sorbed by the sample was calculated based on the weight difference before and after equilibrium.

#### 2.2.11. Drug-Excipient Compatibility Study

The drug-excipient compatibility was investigated using an FTIR spectrophotometer (FTIR-8400S, Shimadzu, Japan). The FTIR spectra of pure paracetamol and its physical mixture with AMTBIS (1 : 1) were scanned in a wavenumber range between 4000 and 500 cm^−1^. A background spectrum was obtained before the samples were run.

#### 2.2.12. Tablet Preparation

Various tablet compacts were prepared using the DC method to investigate compaction, lubricant sensitivity, and dilution potential. Starch compacts with a target weight of 300 mg were formed by applying a compression force that resulted in a crushing strength of 60–80 N for the reference standard (Starch 1500®). All tablets were compressed using a single punch tablet machine (VFD007S21A, Shanghai, China) equipped with a flat-face punch (10 mm in diameter).

*(1) Compaction Property of Starch*. The compaction property was evaluated using the method described by Okunlola and Akingbala [32]. A powder mixture containing 99.5% starch samples and 0.5% magnesium stearate (MgS) was mixed for five minutes and directly compressed into tablets.

*(2) Lubricant Sensitivity*. Lubricant sensitivity was evaluated according to the method used by Assen et al. [17]. A 50 g powder blend, each powder containing 0, 0.25, 0.5, 1, 1.5, and 2% MgS, was mixed for five minutes and compressed into tablets.

*(3) Dilution Potential.* The dilution potentials of the samples were evaluated as described by Shittu et al. [33]. As shown in Table 1, 50 g batches containing different proportions of paracetamol were mixed for ten minutes. Then, MgS was lubricated for five minutes and compressed into tablets.

### 2.3. Tablet Evaluation

#### 2.3.1. Tablet Hardness

The crushing strength (F) was measured for ten randomly selected tablets using a tablet hardness tester (PHARMA TEST, PTB 311E, Germany). The radial tensile strength (TS) was then calculated as described in (4) [29]. The thickness (T) and diameter (D) of each tablet were also measured using the hardness tester.

(4) $$\begin{array}{c} \sigma =\frac{2F}{\pi \;\text{DT}}. \end{array}$$

#### 2.3.2. Friability

Ten tablets of known weight were placed on a friability tester (PHARMA TEST, PTF 10E, Germany) and operated at 25 rpm for 4 min. The tablets were then dedusted and weighed, and their friability was calculated as the percentage of the weight loss of the tablets [29].

#### 2.3.3. Disintegration Test

The disintegration test was performed following the method for uncoated tablets [29]. Six tablets were placed in a disintegration tester (PHARMA TEST, PTZ S, Germany) filled with distilled water at 37 ± 2°C. Then, the time taken for each tablet to fully disintegrate was recorded.

#### 2.3.4. Dissolution Test

The *in vitro* dissolution study was performed using a type II dissolution apparatus (paddle) at a rotation speed of 50 rpm [29]. Six randomly selected tablets were placed in dissolution vessels containing 900 ml of phosphate buffer (pH 5.8) maintained at 37 ± 0.5°C. Then, 10 ml of sample was withdrawn at a defined time interval, properly filtered, and diluted, and then, UV absorbance readings were taken at *λ*_max_ of 243 nm using phosphate buffer (pH 5.8) as a blank.

#### 2.3.5. Data Analysis

Statistical analysis was carried out using analysis of variance (ANOVA) with the SPSS statistical software package (IBM SPSS Statistics 21.0). Tukey's multiple comparison tests were used to compare individual differences. Values were considered statistically significant when *p* < 0.05 at a 95% confidence interval. Origin® 8 software (Origin Pro 8 Corporation, USA) was used to plot the graphs. The results were reported as mean and standard deviation (SD).

## 3. Results and Discussion

### 3.1. Acid Recovery Yield

The acid recovery yield, after 8 days, was 74.16 ± 1.10%. One of the reasons for this weight loss is the hydrolysis of the amorphous regions into shorter water-soluble molecules, which are likely to be removed during the washing process [34]. Compared to Dioscorea and Ethiopian potato starch [19, 21], a higher recovery yield was achieved in the present study. However, the recovery yield was lower compared to Godare starch [20].

### 3.2. Crystallinity of Starch

**Figure 2:**
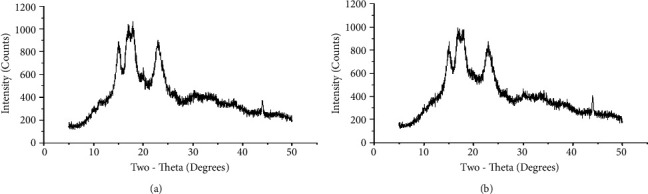
Powder X-ray diffraction pattern of NTBIS (a) and AMTBIS (b).

Figures 2(a) and 2(b) illustrate the XRD patterns of NTBIS and AMTBIS, respectively. Both samples display similar diffraction pattern characteristics of A-type crystals. This is demonstrated by strong peaks at approximately 15.1, 17.0, 17.9, and 23.2° 2*θ* [35]. This suggests that the modification process does not alter the type of crystal [36, 37]. However, the relative crystallinity of the AMTBIS (45.33%) was higher compared to that of the NTBIS (37.87%). This might be due to the hydrolysis of the amorphous regions and extensive reordering of the chain segments [26, 28].

### 3.3. Powder Flow Properties of Starch

Bulk and tap densities increased significantly by acid modification and spray drying individually (*p* < 0.05). However, when combined, the spray-dried AMTBIS showed a greater increase, surpassing all other starches studied (*p* < 0.05). This might be due to the change in the shape and size of the starch granules, which affects their packing arrangement [38]. This finding is in line with previous studies on acid modification [17, 19]. The flow-related properties of NTBIS, AMTBIS, and Starch 1500® are presented in Table 2. AMTBISs exhibited significantly lower CI and HR values compared to NTBISs (*p* < 0.05), indicating improved flow properties. Furthermore, the flow properties of the spray-dried AMTBIS were comparable to those of Starch 1500® (*p* > 0.05). The spray-dried AMTBIS and Starch 1500® demonstrated excellent flowability, with angles of repose measuring 27.10 and 26.53, respectively. However, spray-dried NTBIS had an angle of repose of 36.83, falling within the fair range [29]. NTBIS and AMTBIS dried in the oven did not flow through the funnel, making it impossible to determine their angle of repose and flow rate. This might be due to the presence of irregular and nonspherical particles in higher quantities [39].

### 3.4. Moisture Content and Moisture Sorption Pattern

Dry starch powders typically have a moisture content ranging from 6 to 16%. However, for safe storage, it is recommended to keep it below 13% [40]. In this study, all starches were found to have a moisture content within the recommended range for safe storage. The moisture contents of NTBISs, AMTBISs, and Starch 1500® are presented in Table 2. Consequently, AMTBIS exhibited a significantly lower moisture content compared to NTBIS (*p* < 0.05). This could be attributed to variations in their relative crystallinity. The higher crystallinity of AMTBIS makes it less susceptible to moisture penetration compared to NTBIS.

**Figure 3:**
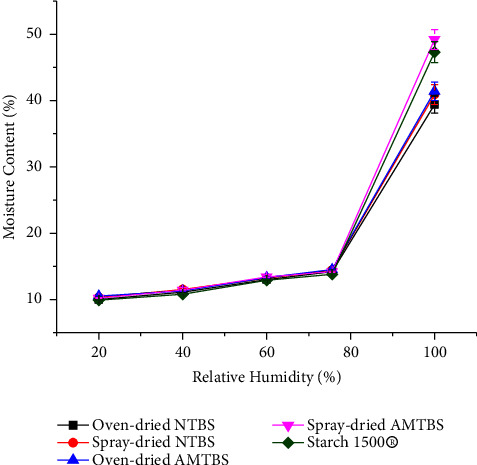
Moisture sorption patterns of NTBIS, AMTBIS, and starch 1500® at different RHs.

The moisture sorption profiles of NTBIS, AMTBIS, and Starch 1500® are depicted in Figure 3. As presented in this figure, the moisture sorbed by the starches ranged between 9.9 and 49.2%. Moisture sorption of all starch samples gradually increased between 20 and 75.6% RH with comparable values (*p* > 0.05). However, beyond 75.6% RH, the moisture sorption profiles of all starch samples showed a significant increase and exhibited a higher moisture sorption value at 100% RH (*p* < 0.05). This higher moisture uptake might be related to the subsequent diffusion of excess moisture into the bulk powder bed [41]. Generally, starch, being a hygroscopic material, requires avoidance of exposure to higher RH values during storage [42].

### 3.5. Swelling Power and Solubility

**Figure 4:**
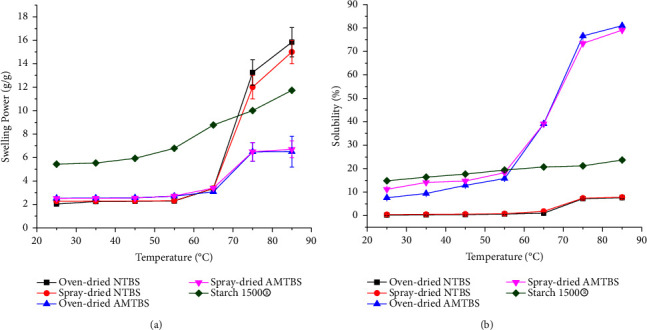
Swelling power (a) and solubility (b) of NTBIS, AMTBIS, and Starch 1500® at different temperatures.

SP and WSI offer information on the extent of interaction between starch chains within the granules [43, 44]. As shown in Figure 4(a), the SP of NTBIS and AMTBIS generally increased with temperature, slightly up to 65°C, and significantly beyond that (*p* < 0.05). Higher temperatures could result in a disruption of the crystalline structure of starch. This exposes OH groups to hydrogen bonding with water molecules, leading to increased granule swelling [45]. However, acid hydrolysis has been shown to reduce swelling power [16]. This might be because it breaks down amorphous regions and increases the crystallinity [46]. Therefore, in line with that, NTBIS showed significantly higher SP than AMTBIS above 65°C (*p* < 0.05).

As shown in Figure 4(b), the WSI of NTBIS, AMTBIS, and Starch 1500® increased with temperature. AMTBISs showed significantly higher WSI at all temperatures compared to NTBISs (*p* < 0.05). This might be attributed to the acid modification that resulted in the formation of partially degraded and shortened chains. This ultimately results in depolymerization and structural weakening of the granules that can enhance the WSI [46–48].

### 3.6. Drug-Excipient Compatibility Study

**Figure 5:**
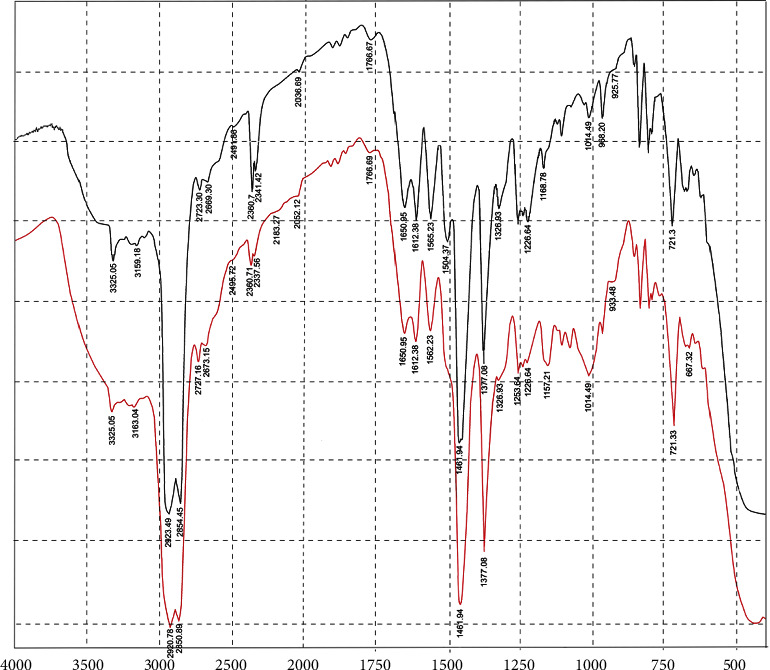
FT-IR spectra of pure paracetamol (black) and its mixture with AMTBIS (red).

Figure 5 shows the FTIR spectra of pure paracetamol and its physical mixture with AMTBIS. Pure paracetamol exhibited characteristic absorption bands at 3325.05 cm^−1^ (O-H stretching), 3159.18 cm^−1^ (N-H stretching), and 1650.95 cm^−1^ (C=O (amide) stretching). Additional bands were also observed at 1562.23 cm^−1^ (amide II stretching), 1226.64 cm^−1^ (-C-O stretching), 3000–2800 cm^−1^ (C-H stretching), and 1612.38 cm^−1^ (aromatic ring stretching vibration). These findings are consistent with previous studies [49, 50]. The presence of these absorption bands in the FTIR spectra of the physical mixture suggests that there is no interaction between paracetamol and AMTBIS.

### 3.7. Compaction Property Study

**Figure 6:**
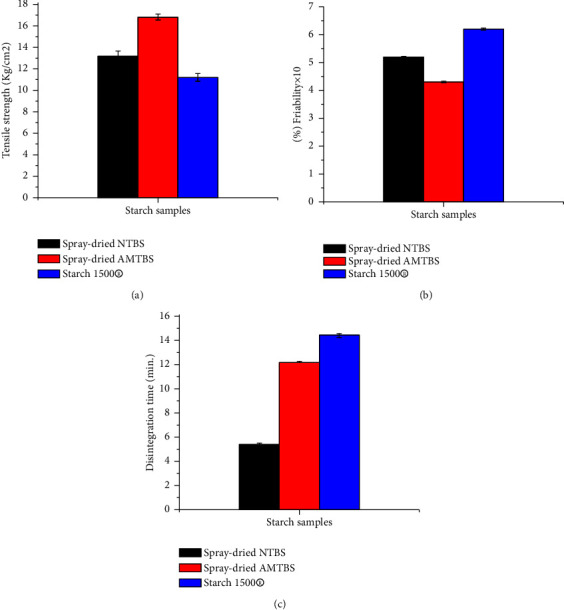
Tensile strength (a), friability (b), and disintegration time (c) of spray-dried NTBIS, spray-dried AMTBIS, and Starch 1500® tablets for compaction study.

In Figure 6(a), it can be observed that the spray-dried AMTBIS exhibited greater compactability compared to the spray-dried NTBIS and Starch 1500® (*p* < 0.05). This can be attributed to the increased relative crystallinity as a result of acid hydrolysis. The improved crystallinity leads to stronger intermolecular forces during compression, resulting in higher TS [15]. These findings are consistent with previous studies conducted on acid-modified starch [31, 51]. The TS of the compacts influences their friability and disintegration time. As shown in Figure 6(b), the friability of tablets of spray-dried NTBIS is greater than that of spray-dried AMTBIS, which is in line with their TS. However, all compacts exhibited a weight loss of less than 1%. As depicted in Figure 6(c), the spray-dried AMTBIS compacts showed a longer disintegration time compared to the spray-dried NTBIS, possibly due to their higher TS. According to Muzikova and Eimerova [52], the extended disintegration time of Starch 1500® tablets, with weaker TS, can be attributed to the development of a gel-like layer on the surface of the tablet.

### 3.8. Lubricant Sensitivity Study

**Figure 7:**
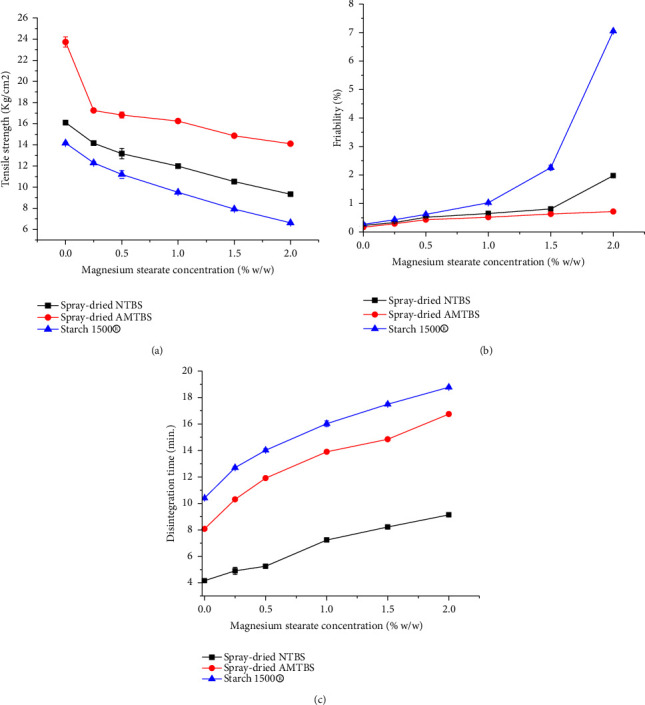
Tensile strength (a), friability (b), and disintegration time (c) of spray-dried NTBIS tablets, spray-dried AMTBIS, and Starch 1500® tablets at different concentrations of magnesium stearate.

The formation of MgS layer around the powder particles reduces the cohesive interactions among them [53]. This can be observed in Figure 7(a), where the TS of the tablets generally decreased with MgS concentration. However, the TS of the tablets made from spray-dried AMTBIS was significantly higher compared to those made from spray-dried NTBIS and Starch 1500® at all MgS concentrations (*p* < 0.05). This indicates that spray-dried AMTBIS has a lower lubricant sensitivity compared to spray-dried NTBIS and Starch 1500®.

Tablets in the study of lubricant sensitivity showed an increase in friability with higher concentrations of MgS, as shown in Figure 7(b). Tablets made from spray-dried AMTBIS had friability below 1% concentrations, making them less friable compared to spray-dried NTBIS and Starch 1500® at all levels of MgS used in the study (*p* < 0.05). Similarly, acetylated TBISs showed lower lubricant sensitivity with acceptable friability of at least 2% MgS level [13], while pregelatinized TBIS [54] was limited to 0.5%. However, this comparison will hold if the different parameters such as compression force were assumed to be similar. On the other hand, the formation of hydrophobic lubricant films around the particles prevents water penetration, wetting, and disintegration of the tablets [55]. Hence, all tablets took longer to disintegrate as the concentration of MgS increased, as shown in Figure 7(c).

### 3.9. Dilution Potential Study

The dilution potential (DP) refers to the amount of API that can be effectively compressed into tablets using a specific DC excipient [56]. To meet the necessary characteristics of the tablet, such as hardness (>50 N) and friability (<1%), the DC excipient should have sufficient DP [57, 58].

#### 3.9.1. Tablet Hardness

**Figure 8:**
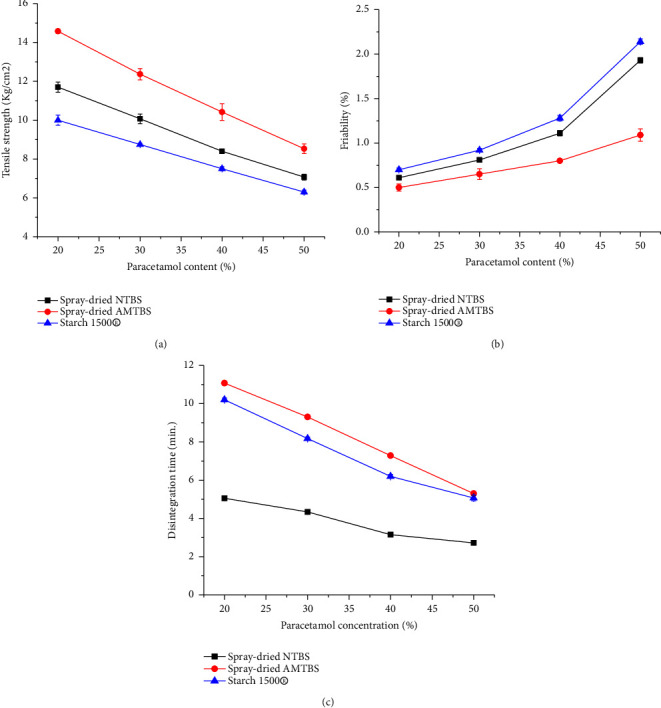
Tensile strength (a), friability (b), and disintegration time (c) of tablets formulated from spray-dried NTBIS, spray-dried AMTBIS, and Starch 1500® at different paracetamol concentrations.

The TS of the tablets used for the DP study decreased with the paracetamol content, as depicted in Figure 8(a). This is due to the poor compactability and the higher elastic recovery of paracetamol [59]. However, tablets from AMTBIS exhibited significantly higher TS compared to NTBIS and Starch 1500® at all paracetamol content. This can be attributed to the higher DP of AMTBIS associated with its superior compactability that overcomes disruptive elastic recovery. Furthermore, tablets made from AMTBIS maintained acceptable hardness (53.5 N) up to 50% paracetamol content, compared to the NTBIS (38.5) and Starch 1500® (37.8 N).

#### 3.9.2. Friability

Figure 8(b) shows that thefriability of the tablets increased significantly with the paracetamol content (*p* < 0.05), corresponding to the decrease in TS. However, the tablets prepared from AMTBIS exhibited lower friability in all paracetamol content and maintained acceptable friability up to 40% paracetamol content. On the other hand, the NTBIS and Starch 1500® were limited to 30%. The dilution potential of AMTBIS is higher compared to a pregelatinized TBIS [54] which was limited to 30% and lower than acetylated TBIS [13], which were acceptable at least 50% paracetamol content. However, these comparisons would provide conclusive information if the different parameters, mainly compression force, were kept similar.

#### 3.9.3. Disintegration Time

Figure 8(c) illustrates that the disintegration time of tablets decreased significantly with the paracetamol content (*p* < 0.05). This can be attributed to the poor compactability of paracetamol, which weakens the tablets and allows water to penetrate the tablet core more easily. However, tablets made from spray-dried AMTBIS exhibited longer disintegration times compared to both spray-dried NTBIS and Starch 1500® at all paracetamol contents. This could be attributed to the higher TS of AMTBIS tablets. Overall, all tablets disintegrated within the acceptable timeframe (<15 minutes) following the pharmacopeial specifications [29].

### 3.10. Study of the Dissolution of Paracetamol Tablets

**Figure 9:**
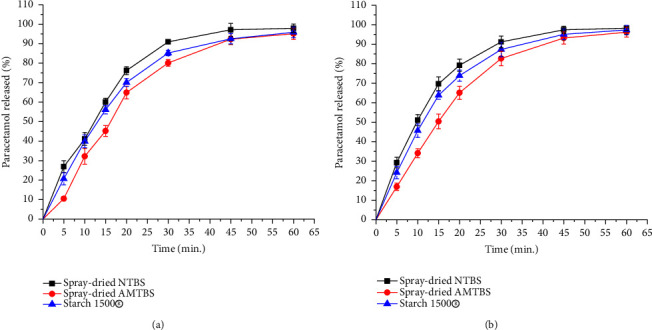
Dissolution profile of tablets prepared from spray-dried NTBIS, spray-dried AMTBIS, and Starch 1500® at 20 (a) and 30% (b) paracetamol contents.

Tablets that passed the evaluations for hardness, friability, and disintegration were selected for the dissolution study. Therefore, tablets containing 20 and 30% paracetamol were selected for each excipient evaluated. As depicted in Figures 9(a) and 9(b), the percentage of drugs released at 30 minutes followed the results of the disintegration time of the tablets. Furthermore, the amount of drug released at 30 minutes generally increased with increasing paracetamol content. This might be attributed to the tablet's weakness with increasing paracetamol content, which facilitated the disintegration of the tablets and hence the dissolution rate. Generally, all paracetamol tablets released more than 80% of their content in 30 minutes, which satisfies the USP specifications for the dissolution of conventional tablets [29].

## 4. Conclusions

The flow properties of NTBIS were significantly improved by acid modification and spray drying. Furthermore, the compactability study showed that AMTBIS had better compactability compared to both NTBIS and Starch 1500®, as indicated by the TS of their compacts. This allowed AMTBIS to successfully incorporate up to 40% of the poorly compressible paracetamol while maintaining acceptable tableting performance. Furthermore, AMTBIS showed a lower lubricant sensitivity compared to the other excipients, even when containing up to 2% MgS, while still maintaining acceptable tableting performance. Overall, based on the results of this study, it can be concluded that spray-dried AMTBIS, specifically the spray-dried version, has the potential to serve as an alternative DC excipient.

### 4.1. Limitations of the Study

The compaction characteristics of the AMTBIS were not studied at different levels of compression forces due to the limitation of the compression machine, which does not read compression forces.

## Figures and Tables

**Table 1:** Paracetamol tablet formulations used for the study of dilution potential.

Ingredients (%)	Formulation											
	F1	F2	F3	F4	F5	F6	F7	F8	F9	F10	F11	F12
Paracetamol	20	30	40	50	20	30	40	50	20	30	40	50
NTBIS (Sd)	75.5	65.5	55.5	45.5	—	—	—	—	—	—	—	—
AMTBIS (Sd)	—	—	—	—	75.5	65.5	55.5	45.5				
Starch 1500®	—	—	—	—	—	—	—	—	75.5	65.5	55.5	45.5
CC Na	4	4	4	4	4	4	4	4	4	4	4	4
MgS	0.5	0.5	0.5	0.5	0.5	0.5	0.5	0.5	0.5	0.5	0.5	0.5

NTBIS: native Taro Boloso-I starch, AMTBIS: acid-modified Taro Boloso-I starch, Sd: spray dried, CC Na: croscarmellose sodium, MgS: magnesium stearate.

**Table 2:** Density and powder flow properties of NTBIS, AMTBIS, and Starch 1500®.

Powder properties	Oven-dried NTBIS	Spray-dried NTBIS	Oven-dried AMTBIS	Spray-dried AMTBIS	Starch 1500®
Bulk density (g/mL)	0.39 ± 0.007	0.47 ± 0.005	0.49 ± 0.008	0.60 ± 0.004	0.61 ± 0.005
Tapped density (g/mL)	0.53 ± 0.003	0.61 ± 0.005	0.56 ± 0.006	0.68 ± 0.005	0.69 ± 0.000
Hausner's ratio	1.35 ± 0.023	1.30 ± 0.021	1.14 ± 0.024	1.12 ± 0.014	1.12 ± 0.008
Carr's index (%)	26.01 ± 1.23	23.27 ± 1.28	12.37 ± 1.83	10.86 ± 1.11	10.70 ± 0.67
Angle of repose (°)	^*∗*^	36.83 ± 0.87	^*∗*^	27.10 ± 0.66	26.53 ± 1.16
Flow rate (g/sec)	^*∗*^	2.11 ± 0.06	^*∗*^	5.48 ± 0.03	10.41 ± 0.28
Moisture content (%)	12.17 ± 0.02	10.16 ± 0.01	11.63 ± 0.03	8.07 ± 0.05	10.65 ± 0.01

^*∗*^The angle of repose and flow rate could not be determined.

## Data Availability

The article contains all the data necessary to support the findings of this study.
